# Patient Profiles of Buprenorphine Initiators in General Healthcare Settings: A Latent Class Approach

**DOI:** 10.1007/s11606-026-10221-z

**Published:** 2026-02-02

**Authors:** Richard A. Grucza, Joanne Salas, Kevin Y. Xu, Jennifer K. Bello, Sarah C. Gebauer, Kirti Veeramachaneni, Fred Rottnek, Jeffrey F. Scherrer

**Affiliations:** 1 Department of Family and Community Medicine, Saint Louis University School of Medicine, St. Louis, MO USA; 2 Department of Health and Clinical Outcomes Research, Saint Louis University School of Medicine, St. Louis, MO USA; 3 Advanced HEAlth Data (AHEAD) Research Institute, Saint Louis University School of Medicine, St. Louis, MO USA; 4 Department of Psychiatry and Behavioral Neuroscience, Saint Louis University School of Medicine, St. Louis, MO USA; 5 Division of Addiction Science Prevention, and Treatment Research, Department of Psychiatry, Washington University School of Medicine, St. Louis, MO USA; 6 SSM Heath, St. Louis, MO USA

**Keywords:** Buprenorphine, opioids, opioid use disorder, substance use disorder, mental health, chronic pain, comorbidity

## Abstract

**Background:**

Buprenorphine is the most commonly used medication for treating opioid use disorder (OUD). It may also be beneficial for individuals with chronic pain who are physically dependent on opioids but do not meet the criteria for OUD. Therefore, the buprenorphine patient population may be heterogeneous in terms of OUD and comorbidities.

**Objectives:**

To characterize the heterogeneity of patient profiles among those initiating buprenorphine treatment in a large multi-state healthcare system, focusing on comorbid pain, psychiatric, and substance use disorder diagnoses, and other patient characteristics associated with profiles.

**Design:**

Retrospective cohort study using electronic health record data.

**Partcipipants:**

Patients with at least 12 months of data before a new buprenorphine prescription (OUD formulation) and at least 6 months of follow-up.

**Main Measures:**

Presence vs absence of opioid use disorder diagnoses and latent class profiles of comorbid diagnoses and clinical characteristics. Post-index outcomes, including the number of buprenorphine orders and incidence of drug-related poisoning, were assessed for each class.

**Key Results:**

In a cohort of 5726 individuals (mean age 38.6 years, 56.2% female, 84.0% white), four latent classes were identified: (1) high multimorbidity burden; (2) moderate multimorbidity burden; (3) high rates of comorbid pain diagnoses but lower rates of psychiatric and substance use disorder diagnoses, with most patients lacking opioid use disorder diagnoses; (4) lower rates of pain diagnoses but high rates of comorbid psychiatric and substance use disorder diagnoses. Post-index measures aligned well with comorbidity profiles.

**Conclusion:**

Patients receiving buprenorphine in general healthcare settings likely form a heterogeneous group. This includes a subgroup with low rates of opioid use disorder diagnoses (class 2, pain comorbidity), who may resemble chronic pain patients more than conventional OUD patients.

**Supplementary Information:**

The online version contains supplementary material available at 10.1007/s11606-026-10221-z.

## INTRODUCTION

Buprenorphine is the most commonly used medication for opioid use disorder (OUD) treatment.^1,2^ Originally approved as an analgesic, doses for OUD formulations are higher than those for pain treatment, with analgesic doses measured in micrograms compared to milligram doses for OUD maintenance.^3–5^ Nonetheless, OUD formulations may be appropriate for individuals who are physically dependent on opioids from long-term pain therapy but do not meet OUD criteria.^6^ Such off-label use is not well characterized, but may be relatively common.^7,8^ Recently, the US Departments of Veterans Affairs (VA) and Defense (DoD) issued guidelines for chronic pain management recommending the use of buprenorphine over full opioid agonists because of its reduced overdose and misuse risks.^9^

Some investigators have proposed that patients who are physically dependent on opioids but do not meet other OUD criteria are best described as having complex persistent opioid dependence (CPOD; or simply “POD” to avoid confusion with other clinical syndromes).^10^ In this putative syndrome, chronic opioid use begins and persists in the context of pain treatment. Dependence occurs without consequences related to opioid misuse or progression to illicit use but raises the risk of unsuccessful taper and withdrawal-related distress.^10–13^ Buprenorphine is increasingly accepted as the likely safest option for such patients.^9,14^ Thus, the population of patients receiving buprenorphine is likely heterogenous and includes both OUD maintenance and POD-like patients transitioning from full agonists who may not have documented OUD diagnoses. However, there is little empirical characterization of the POD syndrome, and traditional OUD patients also commonly present with chronic pain, along with substance use and psychiatric comorbidities.^15–17^ Accordingly, it is difficult to characterize a hypothesized POD-like patient population using a priori definitions, and a data-driven approach is needed to determine whether distinct clinical patterns can be empirically identified within the patient population receiving buprenorphine in general healthcare settings. Latent class analysis (LCA) is a person-centered, data-driven approach that is well suited to this task because it can identify previously undefined patient groups based on shared comorbidity patterns, allowing examination of whether clinically meaningful patterns, such as those resembling POD, emerge from real-world data.

To provide real-world evidence^18^ about the types of patients maintained with buprenorphine and their clinical outcomes such as medication adherence and risk for adverse events,^19^ we sought to characterize heterogeneity among buprenorphine patients in a large healthcare system using electronic health records. We focused on patients using buprenorphine formulations FDA-approved for OUD regardless of whether they had OUD diagnoses. We aimed to (1) characterize new buprenorphine patients overall and by OUD diagnosis with respect to the prevalence of pain, psychiatric and substance use disorders (SUDs) diagnoses, and other clinical characteristics in the 12 months before buprenorphine initiation; (2) use latent class analysis (LCA) to explore heterogeneity within the buprenorphine patient population based on these characteristics; and (3) describe the demographic correlates, provider specialty, and other variables post-index for LCA-derived classifications (see Supplemental Methods for a brief overview of LCA and its applications).

## METHODS

Our focus was on all patients taking buprenorphine regardless of OUD diagnosis. We excluded patients with cancer diagnoses during baseline or follow-up because opioid prescribing guidelines typically differentiate between cancer and non-cancer patients.^20^ Data were obtained from the Saint Louis University-SSM Health (SLU-SSM) Virtual Data Warehouse (VDW) of electronic health records for inpatient and outpatient encounters from over 5 million SSM Health patients in Wisconsin, Illinois, and Oklahoma and Missouri. Data include International Classification of Diseases (ICD)−9/10 diagnostic codes, Current Procedural Terminology (CPT) codes, pharmacy orders, laboratory orders and results, vital signs, provider and clinic type, and demographics. The Saint Louis University Institutional Review Board determined that research using the VDW is exempt from IRB review because all data are historical and de-identified.

### Eligibility

Qualifying orders included buprenorphine-naloxone and mono-product formulations indicated for OUD treatment. We excluded formulations used for anesthesia or acute pain. The index date was the first qualifying buprenorphine order between 1/1/2009 and 6/30/2024. This window used all available VDW data while allowing for a 12-month lookback to measure baseline characteristics and a 6-month follow-up to measure outcomes. Of 5.9 million patients with clinical encounters, 14,138 adult patients without cancer had at least one buprenorphine order. To ensure activity in the healthcare system, patients were required to have at least one encounter in the year prior to the index order (*n* = 12,286). Additional requirements included no buprenorphine orders during look-back, at least one healthcare encounter during follow-up (*n *= 9101), and a second order within 90 days post-index to reduce detox-only cases (*n* = 6575; see appendix eTable 3 for results with this requirement removed). Patients with missing demographics (*n* = 57) and those receiving orders for only pain formulations of buprenorphine (*n* = 792) were excluded, leaving a final sample size of 5726 (eFigure 1).

### Variables

#### Independent/Classification Variables

We first compared baseline characteristics by OUD diagnosis. LCA was then used to group patients based on baseline comorbidities and results were used to compare outcomes and other characteristics by class membership.

### Demographic and Baseline Variables

Demographic variables included age at index, race (White, Black, Other) and gender (male, female). Ethnicity was not analyzed due to a high degree of missingness. Despite the limited accuracy of EHR race data,^21^ inclusion here is warranted to document potential disparities in outcomes. Baseline variables included physical comorbidities (pain conditions, obesity, and smoking), psychiatric disorders, SUDs, prior history of drug-related poisoning, hepatitis C, and endocarditis. The latter two conditions served as indicators of intravenous drug use as they are common complications of using unsterilized needles.^22^ Comorbidities were measured via ICD-9/10 codes (Appendix eTable 1). Non-cancer pain diagnoses were grouped as arthritis, musculoskeletal pain, back and neck pain, fibromyalgia, neuropathy, and chronic pain not elsewhere specified.^23^ Obesity was based on both diagnostic codes and/or recorded BMI ≥ 30. Current smoking was based on ICD-9/10 codes or self-reported “current smoker.” Psychiatric diagnoses included attention deficit hyperactivity disorder (ADHD), bipolar disorder or mania, schizophrenia, major depressive disorder, other mood disorder (dysthymia or mood disorder not otherwise specified), generalized anxiety disorder (GAD), post-traumatic stress disorder (PTSD), and other anxiety disorders (obsessive compulsive disorder, social phobia, panic disorder, or anxiety not otherwise specified). SUDs were defined as ICD-9/10 diagnoses of substance abuse or dependence, including opioid use disorder, alcohol use disorder, and other drug use disorders. ICD 9 and 10 do not define “Opioid use disorder” per se, but recognizing evolving and less pejorative terminology, we will describe these as “use disorder” diagnoses hereafter. Finally, prescription opioid use was defined as having at least one non-buprenorphine opioid prescription during baseline.

### Index and Post-Index Measures

Variables measured at index or during follow-up included physician specialty associated with the index buprenorphine order (psychiatry, pain management, primary care, obstetrician-gynecologist (OBGYN), other, or unknown); whether the patient received buprenorphine monoproduct, buprenorphine-naloxone, or both; the number of buprenorphine orders received (2–4, ≥ 5); whether patients experienced a drug-related poisoning as measured by ICD-9/10 codes; and for patients who received opioid prescriptions during the baseline period, whether they had opioid prescriptions during follow-up.

### Analytic Approach

Analyses were conducted using SAS v9.4 (SAS Institute, Cary, NC). Demographic and baseline characteristics were compared between patients with and without OUD using chi-square tests, independent samples *t*-tests (for age), and Fisher’s exact tests for small cell sizes. Baseline comorbidity variables were used to classify patients into mutually exclusive comorbidity profile categories using latent class analysis (LCA).^24,25^ LCA was performed using Mplus v8.8 (Muthen & Muthen, Los Angeles, CA). Models were fitted stepwise, starting with one latent class. Best fit was assessed via standard fit indices (BIC, adjusted BIC, entropy, Lo–Mendell–Rubin test) and clinical interpretability.^26,27^ After the best-fitting model was chosen, participants were grouped into classes based on predicted class membership probability. Demographic and outcome variables were compared between classes using methods similar to those for baseline characteristics.

## fvRESULTS

Table 1 summarizes baseline characteristics of the cohort overall and by opioid use disorder diagnosis (OUD+, OUD−). Of 5726 patients receiving new buprenorphine prescriptions, approximately 27% lacked baseline OUD diagnoses. The cohort’s mean age was 38.6 years (SD = 13.2); the cohort was predominantly White (84.0%) and female (56.2%). Compared to OUD+ patients, OUD− patients were 6.8 years older (mean age = 43.5 vs. 36.7; *p* < 0.0001), and more likely White (87.2% vs. 82.8%; *p* = 0.0002). OUD– patients had higher prevalence of pain conditions and obesity; OUD + patients had 1.6 to 2.3 times higher rates of psychiatric diagnoses. Alcohol and other drug use disorders were over twice as prevalent in OUD+ patients. Endocarditis, HIV, and prior drug-related poisoning were 1.6 to 2.6 times more prevalent in OUD + patients while hepatitis C was over four times more prevalent in OUD+ patients. OUD− patients were more likely to have received opioid prescriptions during baseline.

**Table 1:** Baseline^a^ Characteristics by OUD Diagnosis, Among Patients Receiving New Prescriptions for Buprenorphine (*n*=5726)

*n* (%) or mean (± sd)	Total (*n* = 5726)	No OUD (*n* = 1558)	OUD (*n* = 4168)	*p*-value^b^
*Demographic variables*				
Age, mean (± sd)	38.6 (± 13.2)	43.5 (± 15.2)	36.7 (± 11.8)	< 0.0001
Race				
White	4809 (84.0)	1359 (87.2)	3450 (82.8)	0.0002
Black	741 (12.9)	162 (10.4)	579 (13.9)	
Other	176 (3.1)	37 (2.4)	139 (3.3)	
Female gender	3216 (56.2)	881 (56.5)	2335 (56.0)	0.722
*Physical comorbidities*				
Arthritis	1396 (24.4)	509 (32.7)	887 (21.3)	< 0.0001
Musculoskeletal pain	1443 (25.2)	511 (32.8)	932 (22.4)	< 0.0001
Back and neck pain	1747 (30.5)	705 (45.3)	1042 (25.0)	< 0.0001
Fibromyalgia	263 (4.6)	124 (8.0)	139 (3.3)	< 0.0001
Neuropathy	345 (6.0)	116 (7.5)	229 (5.5)	0.006
Chronic pain	1281 (22.4)	525 (33.7)	756 (18.1)	< 0.0001
Three or more pain conditions	1048 (18.3)	466 (29.9)	582 (14.0)	< 0.0001
Obesity	1071 (18.7)	436 (28.0)	635 (15.2)	< 0.0001
Smoking	3656 (63.9)	836 (53.7)	2820 (67.7)	< 0.0001
*Psychiatric/substance comorbidities*				
ADHD	314 (5.5)	44 (2.8)	270 (6.5)	< 0.0001
Bipolar/mania	999 (17.5)	185 (11.9)	814 (19.5)	< 0.0001
Major depressive disorder	2058 (35.9)	371 (23.8)	1687 (40.5)	< 0.0001
Other mood disorder	188 (3.3)	31 (2.0)	157 (3.8)	0.001
Schizophrenia	275 (4.8)	53 (3.4)	222 (5.3)	0.002
GAD	880 (15.4)	160 (10.3)	720 (17.3)	< 0.0001
PTSD	320 (5.6)	53 (3.4)	267 (6.4)	< 0.0001
Other anxiety	1298 (22.7)	241 (15.5)	1057 (25.4)	< 0.0001
Alcohol abuse/dependence	791 (13.8)	118 (7.6)	673 (16.2)	< 0.0001
Other drug abuse/dependence	2529 (44.2)	328 (21.1)	2201 (52.8)	< 0.0001
*Additional baseline characteristics*				
Hepatitis C	707 (12.4)	55 (3.5)	652 (15.6)	< 0.0001
Endocarditis	59 (1.0)	7 (0.5)	52 (1.3)	0.007
Drug-related poisoning	738 (12.9)	141 (9.1)	597 (14.3)	< 0.0001
Opioid RX (any)	2253 (39.4)	871 (55.9)	1382 (33.2)	< 0.0001

^a^Occurring in the 12-months prior to or concurrent with index prescription

^b^Independent samples *t*-test, chi-square test, or Fisher’s exact (Monte Carlo estimation) where appropriate

Fit indices for the LCA are shown in e-Table 2. Based on these indices and class interpretability, we chose a four-class solution based on the non-significance of the adjusted Lo-Mendell-Rubin likelihood ratio test comparing a five- to four-class solution (*p* = 0.159). Table 2 lists estimated predicted class probabilities, prevalence of disorders conditional on class membership, and observed prevalences for the full cohort. (see eTable 3 for sensitivity analysis described in “Methods”). We labeled the four classes “moderate multimorbidity” (39.6%), “complex multimorbidity” (19.2%), “pain comorbidity” (15.8%), and “psychiatric and SUD comorbidity” (25.4%). The moderate multimorbidity class had a conditional OUD prevalence (70.5%) that mirrored the overall sample (72.8%) and the lowest rates of baseline opioids, endocarditis, prior drug-related poisoning, musculoskeletal and neuropathic pain, and psychiatric diagnoses. The complex multimorbidity group had a higher prevalence of OUD (86.2%) than the full cohort and the highest rates for most psychiatric diagnoses, and high rates of pain diagnoses, smoking, and baseline opioid prescriptions (65.5%). They also had the highest prevalence of HIV, endocarditis, and prior drug-related poisoning. The pain comorbidity class had high pain and obesity rates but low rates of psychiatric diagnoses, SUDs, and smoking. They had the lowest prevalence of OUD (31.7%), high baseline prevalence of prescription opioids (75.3%), and low prevalence of hepatitis C, HIV, and endocarditis. Conversely, the psychiatric and SUD comorbidity class had the highest rate of OUD diagnoses (91.9%) and low rates of pain diagnoses, but high rates of psychiatric and SUD diagnoses and smoking.

**Table 2:** Results of Latent Class Analysis of Buprenorphine Patients (*n* = 5726) with Four-Class Solutions: Estimated Probabilities

Baseline characteristic	Class 1 (25.4%) “Psych/SUD comorbidity”	Class 2 (15.8%) “Pain comorbidity”	Class 3 (19.2%) “Complex comorbidity”	Class 4 (39.6%) “Moderate comorbidity”	Full cohort
Opioid abuse/dependence	91.9%	31.7%	86.2%	70.5%	72.8%
Arthritis	3.4%	62.5%	54.5%	8.0%	24.4%
Musculoskeletal pain	8.3%	57.6%	57.0%	7.7%	25.2%
Back/neck pain	7.5%	85.9%	57.5%	10.0%	30.5%
Fibromyalgia	0.6%	14.8%	8.0%	1.4%	4.6%
Neuropathy	2.2%	14.1%	13.9%	1.4%	6.0%
Chronic pain	2.1%	70.6%	44.8%	5.2%	22.4%
Obesity	9.1%	51.6%	21.6%	10.3%	18.7%
Smoking	84.6%	37.8%	73.9%	56.1%	63.9%
ADHD	7.5%	3.0%	9.9%	3.0%	5.5%
Bipolar/mania	30.0%	7.3%	35.9%	4.5%	17.5%
Depression	57.4%	18.6%	59.7%	17.6%	35.9%
Schizophrenia	8.3%	0.9%	12.1%	0.6%	4.8%
GAD	23.3%	10.3%	37.5%	0.2%	15.4%
Other mood disorder	6.1%	1.4%	6.6%	0.6%	3.3%
PTSD	9.3%	1.9%	15.2%	0.0%	5.6%
Other anxiety disorder	30.6%	18.4%	48.4%	6.8%	22.7%
Alcohol abuse/dependence	22.4%	2.9%	27.8%	6.0%	13.8%
Other abuse/dependence	76.0%	4.7%	70.4%	26.8%	44.2%
Opioid RX	23.4%	75.3%	65.5%	22.5%	39.4%
Hepatitis C	22.2%	1.7%	22.0%	5.6%	12.4%
Endocarditis	0.8%	0.3%	3.4%	0.3%	1.0%
Drug-related poisoning	17.3%	5.8%	30.8%	4.2%	12.9%

Results from bivariate tests assessing the association of LCA class membership with follow-up outcomes and demographics are shown in Table 3. The psychiatric and SUD comorbidity group was the youngest (mean age = 33.9), followed by the moderate comorbidity (mean age = 35.6), complex comorbidity (mean age = 41.1), and pain comorbidity groups (mean age = 50.9). The pain comorbidity group was more White (90.7%) and female (63.0%) compared to other groups. This group also had the most recent median index year (2020, IQR: 2018, 2022), with other groups differing by at least 1 year on the median and 3–8 years at the 25th percentile. The pain comorbidity group was the most likely to initiate buprenorphine with a primary care (32.6%) or pain management (41.6%) provider while the psychiatric and SUD comorbidity group most likely initiated with psychiatry (61.5%), compared to all other groups. The moderate comorbidity (12.0%) and psychiatric and SUD comorbidity (11.6%) groups most likely initiated with OBGYN, compared to other groups. The moderate comorbidity (48.5%) and pain comorbidity (46.0%) had the highest likelihood of having 5+ buprenorphine prescriptions compared to the complex comorbidity (42.6%) and psychiatric and SUD comorbidity (41.9%) groups. Drug-related poisoning was most common in the complex comorbidity (14.0%) and psychiatric and SUD comorbidity (11.1%), in contrast to pain comorbidity (5.7%) and moderate multimorbidity (4.2%) classes.

**Table 3:** Post-Index Outcomes and Other Correlates by LCA Class Membership (*n* = 5726)

	Class 1 (25.4%) “Psych/SUD comorbidity”	Class 2 (15.8%) “Pain comorbidity”	Class 3 (19.2%) “Complex comorbidity”	Class 4 (39.6%) “Moderate comorbidity”	*p*-value*
*n* (%) or mean (± sd)	1427 (24.9)	890 (15.5)	1062 (18.6)	2347 (41.0)	
*Demographic variables*					
Age, mean (± sd)^b^	33.9 (± 9.9)_1_	50.9 (± 13.8)_2_	41.1 (± 12.4)_3_	35.6 (± 11.9)_4_	< 0.0001
Race					
White	1154 (80.9)	807 (90.7)	865 (81.5)	1983 (84.5)	< 0.0001
Black	224 (15.7)	60 (6.7)	166 (15.6)	291 (12.4)	
Other	49 (3.4)	23 (2.6)	31 (2.9)	73 (3.1)	
Female gender	779 (54.6)	561 (63.0)	555 (52.3)	1321 (56.3)	< 0.0001
Median year of index (IQR)	2018 (2015, 2021)	2020 (2018, 2022)	2019 (2015, 2022)	2016 (2012, 2020)	< 0.0001
*Specialty—initial buprenorphine prescription*					< 0.0001
Psychiatry	878 (61.5)	93 (10.5)	568 (53.5)	731 (31.2)	
Pain management	0 (0.0)	365 (41.0)	28 (2.6)	39 (1.7)	
Primary care	249 (17.5)	290 (32.6)	281 (26.5)	719 (30.6)	
OBGYN	165 (11.6)	10 (1.1)	34 (3.2)	281 (12.0)	
Other	65 (4.6)	80 (9.0)	83 (7.8)	487 (20.7)	
Unknown	70 (4.9)	52 (5.8)	68 (6.4)	90 (3.8)	
**Outcomes**					
Buprenorphine product					< 0.0001
Bupren. monoproduct	154 (10.8)	484 (54.4)	83 (7.8)	299 (12.7)	
Buprenorphine-naloxone	1144 (80.2)	360 (40.5)	912 (85.9)	1889 (80.5)	
Both	129 (9.0)	46 (5.2)	67 (6.3)	159 (6.8)	
*No. of buprenorphine orders*					0.0002
2–4	829 (58.1)	481 (54.0)	610 (57.4)	1209 (51.5)	
≥ 5	598 (41.9)	409 (46.0)	452 (42.6)	1138 (48.5)	
Drug-related poisoning	159 (11.1)	51 (5.7)	149 (14.0)	99 (4.2)	< 0.0001

^*^One-way ANOVA with Tukey post hoc, chi-square test, or Fisher’s exact (Monte Carlo estimation) where appropriate. Kruskal–Wallis for index-year comparisons

^a^Occurring in the 6 months after index date

^b^Means with similar subscripts are not significantly different

Table 4 summarizes receipt of non-buprenorphine opioid prescriptions during follow-up among patients who received them at baseline. The moderate multimorbidity and psychiatric/SUD groups, who were less likely to receive (non-buprenorphine) opioids at baseline, were also the least likely to receive them during follow-up. About two-thirds of the pain comorbidity group and 43.2% of the complex comorbidity group received opioids during follow-up.

**Table 4:** Opioid Outcome in 6 Months After Index, Among Patients with Any Opioid Use at Baseline (*n* = 2253)

*n* (%)	Class 1 (25.4%) “Psych/SUD comorbidity”	Class 2 (15.8%) “Pain comorbidity”	Class 3 (19.2%) “Complex comorbidity”	Class 4 (39.6%) “Moderate comorbidity”
Opioid rx during baseline	*323 (14.3)*	*684 (30.4)*	*711 (31.6)*	*535 (23.8)*
No rx after index (except bupren.)	237 (73.4)	248 (36.3)	404 (56.8)	364 (68.0)
Rx after index	86 (26.6)	436 (63.7)	307 (43.2)	171 (32.0)

*Note*: All comparisons significant at *p*<0.001

## DISCUSSION

Prior LCA-based studies of OUD patients have focused on specialty treatment populations or individuals using/misusing prescribed opioids.^28–33^ This study is, to our knowledge, the first to characterize buprenorphine patients in a general healthcare setting. We analyzed 5726 patients from a large healthcare system receiving new buprenorphine prescriptions. A significant portion of these patients lacked recorded opioid use disorder diagnoses in the EHR. While the absence of such diagnoses does not rule out OUD,^34,35^ these patients exhibited higher rates of chronic pain and lower rates of psychiatric and other SUD diagnoses than those with recorded OUD, thus resembling pain patients than OUD patients.

### LCA Class Characteristics and Clinical Correlates

The latent class analysis identified four patient groups. Two groups were defined by overall levels of comorbidity (“moderate” and “complex”), whereas the other two were characterized by specific types of comorbidity, “pain” and “psychiatric/SUD.” The “pain comorbidity” group was characterized by high rates of pain diagnoses and low psychiatric and SUD comorbidities, including OUD, and initiation of buprenorphine by pain specialists. Based on their low rates of intravenous drug use, drug poisoning, and OUD diagnoses, we speculate that many in this group exhibit physical dependence— a near-universal result of regular opioid use^36^—without OUD, which involves symptoms such as craving, loss of control, and hazardous use. They may most closely resemble a “persistent opioid dependence” category.^10,11^ On the other hand, the “psychiatric/SUD” comorbidity class exhibited high rates of psychiatric and substance use comorbidities but low pain prevalence. They had the highest rates of complications related to intravenous drug use and low baseline opioid prescriptions, suggesting that many of these patients obtained opioids illicitly. Most (91.9%) had OUD diagnoses recorded in the EHR.

### Outcomes and Other Correlates

Both the moderate multimorbidity and pain comorbidity groups exhibited low rates of drug-related poisoning during follow-up, about one-third of the rates for the complex multimorbidity and psychiatric/SUD groups, who each had rates of slightly over 12%. However, the “pain” group also appeared most likely to revert to full opioid agonist use. Thus, while buprenorphine appears relatively safe for this group, they may require closer clinical management when transitioning from full agonists.^37^ The “pain” group had the highest rates of baseline prescription opioid use and low intravenous drug use indicators and was most likely to receive buprenorphine monoproduct. The year of cohort entry was more recent for this group, suggesting that buprenorphine use for such patients is a more recent development. Notably, this class also had the lowest proportion of non-White patients (9.0% vs. 15.5% for the full cohort), suggesting that disparities in treatment of OUD patients with buprenorphine may also extend to chronic pain patients.^35,38^ This was the smallest identified group, comprising 15.8% of the cohort. This may reflect challenges in transitioning from full agonists. Insurance barriers to off-label use of higher-dose formulations may also impede broader adoption. Such barriers have been documented in OUD maintenance treatment,^39–41^ and may be even more pronounced for off-label prescribing.

The psychiatric/SUD comorbidity group and complex multimorbidity group both had elevated risk for drug-related adverse events (i.e., drug-related poisoning) compared to the “pain” and “moderate” groups. This is consistent with prior research on the association between psychiatric and substance use disorders and drug-related poisoning.^42–44^ However, this should not be taken to indicate that buprenorphine fails to mitigate risk in this group as buprenorphine treatment is associated with lower risks than non-medication approaches even among poly-SUD patients.^19,45^ They were unlikely to be prescribed buprenorphine monoproduct, and no patients in the group were initiated on buprenorphine by a pain specialist.

#### Limitations and Conclusion

Limitations of this study include that the data were from a large Midwestern-Southern healthcare system and results may not generalize to other geographic regions. We do not know the symptoms or patient concerns that prompted the OUD diagnosis or buprenorphine prescription. We did not have lifetime clinical histories to further refine group descriptions. Because we have medication orders data but not pharmacy claims, we do not know if patients filled their buprenorphine prescriptions. Because we only had diagnostic codes, OUD misclassification is possible. Finally, buprenorphine prescribing regulations and practices may have changed over time over time^46–48^ causing changes in prevalence and/or characteristics of the classes we found.

In summary, among patients with new prescriptions for OUD formulations of buprenorphine, we found substantial heterogeneity in diagnoses of opioid use disorder, pain, and psychiatric and substance use comorbidities. Our analysis suggests that this heterogeneity could be by the presence of four clinically interpretable patient groups that vary in comorbidities, buprenorphine adherence, and risk for drug-related poisoning. The identification of a “pain” subgroup, characterized by high rates of pain diagnoses, low rates of OUD diagnoses, and treatment initiation in primary care or pain settings is our most novel finding. Such patients are likely present in many healthcare systems, but this is, to our knowledge, the first study to quantify and characterize this subset in a general healthcare population. Their favorable short-term safety profile but elevated likelihood of returning to full opioid agonists underscores the need for tailored clinical management, clearer tapering strategies, and close follow-up during transitions from long-term opioid therapy.^37^

Our discussion has highlighted several clinical and policy implications that we summarize here. The existence of a pain-comorbidity group suggests that individuals with physical dependence from long-term opioid therapy may benefit from higher-dose buprenorphine formulations typically used for OUD but may face coverage restrictions that impede such care. This group had the lowest proportion of non-White patients, raising concerns that racial disparities in buprenorphine access extend beyond OUD treatment and emphasizing the need for more equitable prescribing practices and insurance policies.^35,38–41^ The increasing size of this group over time further suggests that policy reforms, along with evolving clinical guidelines recommending buprenorphine in a broader set of contexts, may be expanding access. Continued efforts to reduce regulatory and coverage barriers could extend these benefits more broadly.^9,46–48^ The psychiatric/SUD and complex-comorbidity groups demonstrated substantially higher risks for adverse events, even as buprenorphine remains safer than non-medication approaches for high-risk patients. This suggests the potential utility of risk-stratified care, which may include more frequent follow-up visits and medication-counseling. Future research should extend these findings through longer observation periods, assessment of clinical trajectories, and evaluation of how these patient profiles inform treatment engagement, safety, and successful transition to buprenorphine.

## Supplementary Information

Below is the link to the electronic supplementary material.

ESM 1 (DOCX 33.3 KB)

## Data Availability

Due to the proprietary nature of the data, it cannot be shared. Any code used for variable creation and statistical analysis will be made available upon written reasonable request to the corresponding author.
