# Supplementary material for: Patient Profiles of Buprenorphine Initiators in General Healthcare Settings: A Latent Class Approach
Source: J Gen Intern Med. 2026 Feb 2;41(9):2465–73. doi: 10.1007/s11606-026-10221-z (PMC13304395; doi:10.1007/s11606-026-10221-z)
Supplement: Supplementary file 1 — (DOCX 33.3 KB) [file 11606_2026_10221_MOESM1_ESM.docx]

**Supplemental Material**

***Brief overview of latent class analysis.***

LCA is a data-driven, person-centered approach that identifies distinct groups based on patterns of association among observed variables and assumes that these associations are driven by underlying (or latent) patient types, meaning that within each group, the observed characteristics are independent of one another.^1^ In other words, comorbidities and other clinical features are best explained by group membership rather than by other unmeasured factors. With further validation and implementation work, such classifications can support targeted treatment, risk stratification, and outcome-based decision-making.^2,3^ While initial analyses are exploratory, they can reveal meaningful heterogeneity within a patient population and determine whether the identified groups differ in their clinical trajectories and outcomes.^4^

**References for Supplemental Material**

1. Aflaki K, Vigod S, Ray JG. Part I: A friendly introduction to latent class analysis. *Journal of Clinical Epidemiology*. 2022;147:168-170. doi:10.1016/j.jclinepi.2022.05.008

2. Shimshock S, Chor KHB, Brylske PD. Using latent class analysis to identify clinical subgroups and pathways of youth in a therapeutic foster care program. *Children and Youth Services Review*. 2022;141:106626. doi:10.1016/j.childyouth.2022.106626

3. Yang Q, Zhao A, Lee C, Wang X, Vorderstrasse A, Wolever RQ. Latent Profile/Class Analysis Identifying Differentiated Intervention Effects. *Nursing Research*. 2022;71(5):394-403. doi:10.1097/NNR.0000000000000597

4. Weller BE, Bowen NK, Faubert SJ. Latent Class Analysis: A Guide to Best Practice. *Journal of Black Psychology*. 2020;46(4):287-311. doi:10.1177/0095798420930932

| **e-table 1. Diagnostic code variable definitions** | |
| --- | --- |
| **Baseline characteristics – measured in the baseline period (year prior to and including index date) and present if at least one diagnostic code (unless otherwise stated).** | |
| Cancer (exclusionary diagnosis) | ICD9 code: 140.x-209.x  ICD-10 code: C00.x to D09.x |
| Arthritis | ICD-9 code: 710.0, 710.1, 710.2, 710.3, 710.4, 710.8, 710.9, 711.x, 713.x-717.x, 718.0x, 718.1x, 718.2x, 718.3x, 718.5x, 718.6x, 718.7x, 718.8x, 718.9x, 719.x, 720.0, V13.4;  ICD-10 code: M00.x to M02.x, M05.x, M06.x, M08.x, M12.x to M19.x, M23.x, M24.0x to M24.4x, M24.6x to M24.9, M25.x, M32.10, M33.20, M33.90, M34.0, M34.1, M34.9, M35.00, M35.01, M35.5, M35.9, M36.2, M36.3, M36.4, M43.4, M43.5x, M45.9, M79.6x, R26.2, R29.4, R29.898, Z87.39 |
| Musculoskeletal pain | ICD-9 code: 725.x, 726.0, 726.1x, 726.2, 726.3x, 726.4, 726.5, 726.6x, 726.71, 726.72, 726.90, 727.00, 727.03, 727.04, 727.05, 727.06, 727.09, 727.2, 727.3, 727.49, 727.50, 727.51, 727.6x, 727.89, 727.9, 729.0, 729.4, 729.5, 729.7x, 729.89, 729.9, 729.91, 729.92, 781.99, 830.x-848.x, 905.6, 905.7, V43.6x, V43.7. V48.3, V49.6x, V49.7x;  ICD-10 code: M35.3, M60.x to M79.x (**excluding M79.7**), R29.898, R29.91, S03.x, S13.x, S16.x, S23.x, S33.x, S39.0x, S39.9x, S43.x, S46.x, S53.x, S56.x, S63.x, S66.x, S73.x, S76.x, S83.x, S86.x, S93.x, S96.x, Z96.6x, Z97.1x, Z89.x |
| Back and Neck pain | ICD-9 code: 720.1, 720.2, 720.8x, 720.9, 721.x-722.x, 723.0-723.3, 723.5-723.7, 723.9, 724.x, 756.1x;  ICD-10 code: M43.2x, M43.6, M43.8x, M43.9, M46.0x, M46.1, M46.4x, M46.8x, M46.9x, M47.x, M48.0x, M48.1x, M48.2x, M48.3x, M48.8x, M48.9, M49.8x, M50.x to M51.x, M53.x to M54.x, M96.1, Q76.0 to Q76.3, Q76.4x, Q76.6 |
| fibromyalgia | ICD9 - 729.1  ICD10 – M79.7 |
| Chronic pain | ICD-9: 338.2x  ICD-10: G89.2x |
| Neuropathy | ICD-9 code: 053.13, 072.72, 337.0x, 337.1, 353.x-357.x, 377.33, 377.34, 377.41;  ICD-10 code: B02.23, B26.84, G90.0x, G99.0, G54.x to G65.x |
| HIV | ICD-9 code: 042x – 044x  ICD-10 code: B20x – B24x  *measured at any point in record prior to index |
| Obesity | ICD-9 code: 278.00, 278.01, 278.03, V85.3, V85.4  ICD-10 code: E66.9, E66.01, E66.1, E66.2, E66.8, E66.9, Z68.3, Z68.4  BMI ≥ 30 |
| Smoking | ICD-9 code: V15.82, 305.1;  ICD-10 code: Z87.891, Z72.0, F17.20x, F17.21x  Self-reported current smoker in social history |
| ADHD | ICD-9 code: 314.0x  ICD-10 code: F90.x |
| Bipolar/Mania | ICD-9: 296.0x, 296.1x, 296.4x, 296.5x, 296.6x, 296.7x, 296.8x, 298.9x  ICD-10: F30.x, F31.x |
| Major depressive disorder | ICD-9 code: 296.2x, 296.3x, 311;  ICD-10 code: F32.0-F32.5, F32.9, F33.0-F33.3, F33.4x, F33.9  - 2 outpatient occurrences (on different days) in same 12 month period or 1 inpatient occurrence |
| Other mood disorder | *Composite of dysthymia and mood NOS  ICD-9 code: 300.4, 296.9x;  ICD-10 code: F34.1, F39  - 2 outpatient occurrences (on different days) in same 12 month period or 1 inpatient occurrence |
| Schizophrenia | ICD-9 code: 295.x;  ICD-10 code: F20x, F25x |
| GAD | ICD-9 code: 300.02;  ICD-10 code: F41.1  - 2 outpatient occurrences (on different days) in same 12 month period or 1 inpatient occurrence |
| PTSD | ICD-9 code: 309.81;  ICD-10 code: F43.1x  - 2 outpatient occurrences (on different days) in same 12 month period or 1 inpatient occurrence |
| Other anxiety | *Composite of OCD, Anxiety NOS, Social Phobia, Panic Disorder  ICD-9 code: 300.3, 300.00, 300.09, 300.23, 300.01;  ICD-10 code: F42, F41.8, F41.9, F40.1x, F41.0  - 2 outpatient occurrences (on different days) in same 12 month period or 1 inpatient occurrence |
| Alcohol abuse/dependence | ICD-9 code: 303.9x, 305.0x;  ICD-10 code: F10.x |
| Opioid abuse/dependence | ICD-9 code: 304.0x, 305.5x, 304.7x;  ICD-10 code: F11.x |
| Other drug abuse/dependence | * Composite of cocaine, sedative, amphetamine, cannabis, hallucinogen or other  ICD-9 code: 304.1x, 304.2x, 304.3x, 304.4x, 304.5x, 304.6x, 304.8x, 304.9x, 305.2x, 305.3x, 305.4x, 305.6x, 305.7x, 305.9x  ICD-10 code: F12.x, F13.x, F14.x, F15.x, F16.x, F18.x, F19.x |
| Hepatitis C | ICD-9 code: 070.41, 070.44, 070.51, 070.54, 070.7x, V02.62;  ICD-10 code: B17.1x, B18.2, B19.2x |
| Endocarditis | ICD-9 code: 036.42, 098.84, 112.81, 115.04, 115.14, 115.94, 421x, 424.9x;  ICD-10 code: A32.82, A39.51, A52.03, A54.83, B33.21, B37.6, I01.1, I33x, I38, I39, M32.11 |
| ≥1 opioid RX | A prescription for codeine, dihydrocodeine, fentanyl, hydrocodone, hydromorphone, levorphanol, meperidine, methadone, morphine, oxycodone, oxymorphone, pentazocine, tapentadol, ortramadol  *also an outcome measured in the 6-months after index |
| **Outcome variables – measured in the 6-months after index. Presence if at least one diagnostic code.** | |
| Drug-related poisoning | ICD-9 code: 960x – 989x, E850x – E858x, E860x – E869x, E950x – E952x, E962x, E972, E975, E976, E980x – E982x;  ICD-10 code: T40x – T65x |

| eTable 2. Latent class analysis, MPlus v8.8 – relative model fit indices and classification diagnostics for comorbidities among patients initiating BUP (n=5,726) | | | | | | | |
| --- | --- | --- | --- | --- | --- | --- | --- |
| Classes | #par | LL | AIC | BIC | aBIC | Entropy | aLMR, p-value |
| 1 | 24 | -56494.20 | 113036.40 | 113196.06 | 113119.80 | --- | --- |
| 2 | 49 | -53503.86 | 107105.72 | 107431.70 | 107275.99 | 0.79 | <.0001 |
| 3 | 74 | -51915.83 | 103979.66 | 104471.96 | 104236.81 | 0.77 | <.0001 |
| 4 | 99 | -51421.63 | 103041.25 | 103699.88 | 103385.28 | 0.72 | <.0001 |
| 5 | 124 | -51212.01 | 102672.02 | 103496.96 | 103102.93 | 0.75 | 0.159 |
| Note: Bolded model selected as best fitting model.  #par = number of parameters estimated; LL=log likelihood; AIC=Akaike information criterion; BIC=Bayesian information criterion; aBIC=sample size adjusted Bayesian information criterion; aLMR = adjusted Lo-Mendell-Rubin likelihood ratio test comparing solution for k vs. k-1 classes | | | | | | | |

| eTable 3: Results of latent class analysis of buprenorphine patients after removing the “two or more” order restriction. (n=7,473) with four class solution. Estimated probabilities | | | | | |
| --- | --- | --- | --- | --- | --- |
| Baseline Characteristic | Class 1 (18.9%)  “High Comorbidity” | Class 2 (17.7%)  “Pain Comorbidity” | Class 3 (37.9%)  “Low Comorbidity” | Class 4 (25.6%)  “Psych Comorbidity” | Full Cohort |
| Opioid abuse/dependence | 83.5% | 30.3% | 65.3% | 89.6% | 68.8% |
|  |  |  |  |  |  |
| Arthritis | 56.7% | 61.9% | 8.0% | 3.9% | 25.7% |
| Musculoskeletal pain | 59.1% | 56.3% | 8.1% | 8.8% | 26.4% |
| Back/neck pain | 56.6% | 80.5% | 9.4% | 7.3% | 30.3% |
| Fibromyalgia | 7.0% | 13.6% | 1.4% | 1.0% | 4.5% |
| Neuropathy | 13.8% | 13.7% | 1.3% | 2.3% | 6.1% |
| Chronic pain | 46.5% | 67.4% | 4.8% | 1.7% | 23.0% |
|  |  |  |  |  |  |
| Obesity | 22.7% | 51.0% | 10.6% | 8.6% | 19.5% |
| Smoking | 75.8% | 38.1% | 55.5% | 84.3% | 63.6% |
|  |  |  |  |  |  |
| ADHD | 9.7% | 3.5% | 3.2% | 7.9% | 5.7% |
| Bipolar/mania | 37.4% | 7.7% | 4.2% | 29.8% | 17.6% |
| Depression | 58.3% | 19.6% | 16.9% | 57.1% | 35.5% |
| Schizophrenia | 13.4% | 0.8% | 0.7% | 9.0% | 5.2% |
| GAD | 36.7% | 9.8% | 1.8% | 23.6% | 15.4% |
| Other mood disorder | 7.8% | 1.1% | 0.6% | 7.1% | 3.7% |
| PTSD | 15.2% | 1.9% | 0.0% | 9.0% | 5.5% |
| Other anxiety disorder | 48.5% | 18.9% | 7.0% | 31.8% | 23.3% |
|  |  |  |  |  |  |
| Alcohol abuse/dependence | 28.6% | 2.9% | 5.5% | 21.6% | 13.5% |
| Other abuse/dependence | 70.0% | 4.8% | 25.9% | 75.1% | 43.1% |
|  |  |  |  |  |  |
| Opioid RX | 64.4% | 74.9% | 22.3% | 25.1% | 40.3% |
| Hepatitis C | 23.1% | 1.4% | 5.5% | 21.7% | 12.2% |
| Endocarditis | 2.9% | 0.3% | 0.3% | 0.7% | 0.9% |
| Drug-related poisoning | 31.6% | 5.9% | 5.2% | 20.1% | 14.1% |
| Note that the “order of classes” changed but sizes of classes and conditional prevalence of comorbidities did not meaningfully change by relaxing inclusion criteira. | | | | | |

**e-Figure 1. Sampling**

**Base sample: 5,972,796 patients (all ages) with an inpatient (IP), ambulatory (AV), or telehealth (VC) encounter 1/1/2009 – 6/30/24**

BUP prescription (RX) 1/1/09-6/30/24 (n=15,198)

No cancer (n=14,154)

Adults ≥18 years old at BUP RX

(n=14,138)

IP, AV, or VC encounter in year prior to BUP RX (n=12,286)

IP, AV, or VC encounter > 6-months after BUP RX (n=9,909)

New BUP RX – no BUP RX in year prior (n=9,101)

≥ 1 additional BUP RX within 90 days (n=6,575)

Remove missing demographics

(n=6,518) (1 sex, 56 race)

Remove patients w/ only BUP pain formulation in 6-month after new BUP episode (n=5,726)
